# Significance of Plant Growth Promoting Rhizobacteria in Grain Legumes: Growth Promotion and Crop Production

**DOI:** 10.3390/plants9111596

**Published:** 2020-11-17

**Authors:** Karivaradharajan Swarnalakshmi, Vandana Yadav, Deepti Tyagi, Dolly Wattal Dhar, Annapurna Kannepalli, Shiv Kumar

**Affiliations:** 1 Division of Microbiology, ICAR-Indian Agricultural Research Institute (IARI), New Delhi 110012, India; vandana21yadav@gmail.com (V.Y.); kkdeeptityagi@gmail.com (D.T.); dollywattaldhar@yahoo.com (D.W.D.); annapurna96@gmail.com (A.K.); 2 International Centre for Agricultural Research in the Dry Areas (ICARDA), Rabat 10112, Morocco

**Keywords:** grain legumes, rhizobia, PGPR, crop growth, productivity

## Abstract

Grain legumes are an important component of sustainable agri-food systems. They establish symbiotic association with rhizobia and arbuscular mycorrhizal fungi, thus reducing the use of chemical fertilizers. Several other free-living microbial communities (PGPR—plant growth promoting rhizobacteria) residing in the soil-root interface are also known to influence biogeochemical cycles and improve legume productivity. The growth and function of these microorganisms are affected by root exudate molecules secreted in the rhizosphere region. PGPRs produce the chemicals which stimulate growth and functions of leguminous crops at different growth stages. They promote plant growth by nitrogen fixation, solubilization as well as mineralization of phosphorus, and production of phytohormone(s). The co-inoculation of PGPRs along with rhizobia has shown to enhance nodulation and symbiotic interaction. The recent molecular tools are helpful to understand and predict the establishment and function of PGPRs and plant response. In this review, we provide an overview of various growth promoting mechanisms of PGPR inoculations in the production of leguminous crops.

## 1. Introduction

Grain legumes (Family Leguminosae), also called pulses, are high in protein content (20–25%) and form an essential part of daily diets across the globe. The protein-rich grains of these crops are also a good source of vitamins, minerals, prebiotics, and other important nutrients. Globally, pulses are grown on 95.7 million ha area, as rainfed crops, mainly on marginal lands with minimum agro-inputs wherein a diverse range of soil microorganisms play a vital role. Soil microorganisms form an integral part of nutrient cycling processes and are crucial determinants of soil fertility and health. The beneficial soil bacteria which colonize roots and their surroundings (rhizosphere) are collectively called plant growth-promoting rhizobacteria (PGPR) [1]. They form symbiotic, associative or neutral association with plants and have a significant influence on crop growth and development. PGPRs stimulate plant growth by nutrient mobilization, solubilization, and transformation [2,3,4] and protect plants from pathogenic infections [5,6,7]. The colonization potential of PGPRs is driven by chemo-taxis response with root exudates that either attract or deter rhizospheric microorganisms [8,9]. It is estimated that about 30% of plant photosynthates are released via root exudation [10], which consists of high and low molecular weight compounds like sugars, proteins, organic acids, flavonoids, mucilage, etc. [11]. A proportion of the root exudate molecules can be metabolized by rhizobacteria for their own utilization in the immediate vicinity of roots, or can be taken up by plants for growth. Root exuded flavonoids are the key signals for legume-rhizobial and legume-mycorrhizal interactions and their establishment [12]. Rhizobia along with other PGPRs inhabit the roots of legumes, which can directly improve plant growth through their influence on physiological and biochemical parameters of the host. Hence, the present review attempts to understand the role of PGPRs and their applications in leguminous crops.

## 2. Growth-Promoting Mechanisms of PGPR

**Figure 1:**
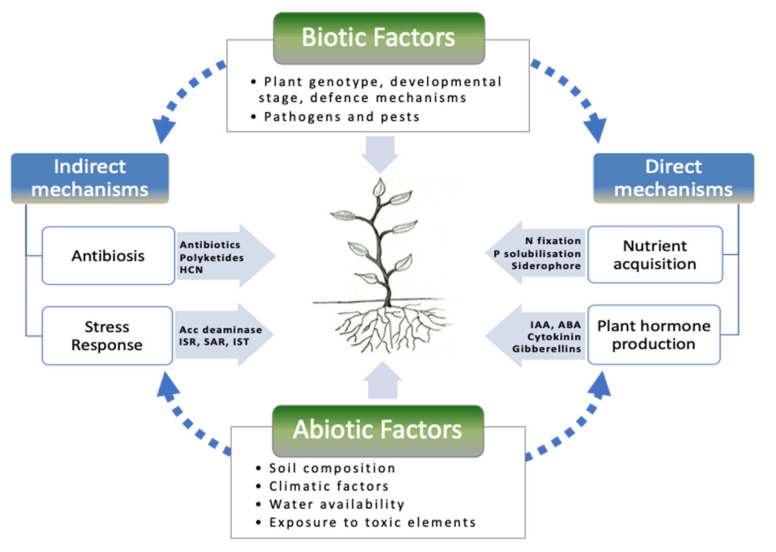
Biotic and abiotic factors influencing plant– plant growth promoting rhizobacteria (PGPR) interactions in the rhizosphere.

Plant growth promotion by rhizobacteria can occur directly or indirectly at different times during the life cycle of the plant [13]. Direct growth promotion includes nitrogen fixation [14], phosphate solubilization [15], phytohormone production [16] or enhancement in the availability of minerals [17]. The N fixation process is mediated by an oxygen-sensitive, nitrogenase enzyme complex which converts the atmospheric nitrogen into an ammonical form (biologically fixed nitrogen) that is either made available to the plants or released in the soil. Phosphate solubilizers mobilize fixed forms of phosphorus already present in the soil in the available form to the plant. The production of plant hormones such as auxins, gibberellins and cytokinins also influence plant growth. Production of siderophores by PGPR helps the plant with enough iron in iron-limited soils. Other beneficial effects on plant growth attributed to PGPR include osmotic adjustment, stomatal regulation, modification of root morphology, etc. under abiotic stress conditions [18,19]. Indirect growth promotion of PGPR is attributed to the prevention of the deleterious effects of phytopathogens [5] by producing antagonistic substances such as phenazine, diacetylphloroglucinol (DAPG), hydrogen cyanide (HCN), 2–3 butanediol, acetoin [20] and siderophores [21]. The lytic enzymes *viz*., chitinase and glucanase produced by these PGPRs can degrade the cell-wall of fungal pathogens, thus inducing systematic resistance throughout the entire plant system [22]. However, the ways by which PGPRs influence plant growth directly may differ from species to species or can be strain specific. The positive effects of PGPR inoculations have been studied in many plants, and Table 1 enlists some of the examples where these bacteria have significantly enhanced the growth and development of legumes. PGPRs interact with plants through various direct and indirect mechanisms which are functions of PGPR activities and biotic as well as abiotic factors present in the surroundings (Figure 1).

### 2.1. Nitrogen Fixation

Diazotrophic microorganisms fix atmospheric nitrogen either as free-living or in symbiotic association with higher plants. N requirement for sustained productivity of pulses relies on symbiotic nitrogen fixation (SNF) by root nodulating bacteria called rhizobia. The genetic and metabolic integrity of rhizobia imparts ecologically effective adaptation to legume crops under nitrogen-depleted soil [59]. These organisms are Gram-negative, rod-shaped motile, non-spore forming and live freely in the soil, showing chemoheterotrophic mode of nutrition with G+C content of 59–65.5%. They have an ability to produce extracellular polysaccharides of varying compositions and exhibit slimy growth on YEMA (yeast extract mannitol agar) medium. Taxonomical studies on rhizobia gave the theory of cross-inoculation groups in which rhizobia isolated from one plant can nodulate other plants of the same group [60]. Later, fast-growing *Rhizobium* and slow-growing *Bradyrhizobium* were reported on the basis of their growth on laboratory media [61]. Rhizobial strains isolated from pea, bean and clover are known as fast growers, whereas those isolated from soybean and cowpea are characterized as slow growers. *Mesorhizobium* species that nodulates a wide range of hosts including acacia, astragalus, chickpea, lotus, lupinus, leucaena, prosopis, etc. show characteristics of intermediate growth rates [62]. On the basis of 16S rRNA gene sequence, rhizobia have been divided into six genera namely, *Azorhizobium*, *Allorhizobium*, *Bradyrhizobium*, *Mesorhizobium*, *Rhizobium*, and *Sinorhizobium* [63], with a strong specificity between leguminous hosts and nodulating rhizobial strains [60]. According to current taxonomic classification, 14 genera and 98 species have been identified in rhizobia belonging to diverse groups such as α-proteobacteria (*Azorhizobium*, *Bradyrhizobium*, *Mesorhizobium*, *Rhizobium*, *Ensifer*, *Phyllobacterium*, *Microvirga*, *Ochrobactrum*, *Methylobacterium*, *Devosia and Shinella*), β-proteobacteria (close to *Burkholderia*, *Cupriavidus* (formerly *Ralstonia*) and γ-proteobacteria (*Pseudomonas*) [64]. The complex interaction between rhizobia and host legumes is mediated by plant signals, particularly flavonoids, which in turn can activate nodulation genes (*nod/nol/noe*) and synthesize Nod factor, which is a host determinant in rhizobia [65]. Rhizobia form two types of nodules, determinate and indeterminate [66]. Determinate nodules are spherical due to early meristem termination and are found in soybean, common bean, *Lotus*, and *Vigna* species, whereas indeterminate nodules are cylindrical in shape due to later meristem termination, and are found in pea, alfalfa, clover and vetch. Different strains of rhizobia can fix atmospheric nitrogen into ammonia with the help of enzyme nitrogenase.

Nitrogenase N_2_ + 8H^+^ + 8e^−^ + 16 ATP → 2NH_3_ + H_2_ + 16ADP + 16 Pi

The *nif* and *fix* genes are involved in symbiotic nitrogen fixation [67] and the symbiotic effectiveness of different legumes varies depending on the host and rhizobial strains. It is estimated that 100–175 million metric tons of nitrogen is fixed through the biological nitrogen fixation process [68], in which SNF contributes 70 million metric tons annually [69] or 24 to 584 kg N ha^−1^ yr^−1^ [70]. SNF also offers organic nitrogen that becomes slowly available to non-legume crops [71]. Legume–rhizobial symbiosis alone fulfils the one-third of the global N demand. The amount of nitrogen fixed as a result of SNF by rhizobia is summarized in Table 2. Besides rhizobia, some non-rhizobial nodule inhabiting bacteria such as *Arthrobacter*, *Bacillus*, *Burkholderia*, *Dyella*, *Methylobacterium*, *Microbacterium*, *Staphylococcus* and *Streptomyces* isolated from legume root nodules are reported to possess plant growth, promoting activities such as nitrogen fixation, P solubilization and growth promotion [72,73,74]. In addition to root nodulating bacteria, other free-living diazotrophic bacteria such as *Azotobacter*, *Arthrobacter*, *Acinetobacter*, *Bacillus*, *Burkholderia*, *Enterobacter*, *Erwinia*, *Azospirillum*, *Acetobacter*, *Azoarcus*, *Beijerinckia*, *Herbaspirillum*, and *Gluconacetobacter* isolated from rhizosphere soil can also contribute up to 36 kg N ha^−1^ year^−1^ [70].

### 2.2. P Solubilization

Phosphorus is one of the macronutrients essential for legume growth and symbiotic nitrogen fixation. P application, along with *Rhizobium tropici* inoculation, resulted in an increase of plant parameters in *Phaseolus vulgaris*. There was also an enhanced effect on nodulation and N fixation with a 20-fold increase in ARA (acetylene reduction assay) activity with P application [89]. Phosphorus is required for nodule initiation, its development and functioning, along with the whole plant growth [90]. Application of low phosphorus markedly affected plant growth and SNF in soybean while an increase in P enhanced whole plant N associated with an increase in the number of nodules and nodule mass. Co-inoculation with P solubilizer along with rhizobia resulted in increased growth, nodulation and grain yield in common bean [17] and chickpea [91,92] in comparison to control.

In spite of the abundance of phosphorus in organic and inorganic forms in the soils, the available P remains low. When P is applied to the soil, it gets rapidly fixed, resulting in low P availability for the plants. As a result, a large proportion of P in soil is in insoluble form and only a small proportion gets immediately available to plants. Since the world reserves of non-renewable P rocks are becoming increasingly scarce and geologic P deposits will get depleted in 50–100 years [93], the application of P solubilizing microorganisms (PSMs) has shown potential in the transformation of unavailable forms of phosphorus to available form, which, in turn, can help in reducing the escalating price of rock phosphate due to fast depletion of its reserves.

Conversion of insoluble phosphates to orthophosphate by PSMs is an important PGPR trait, which can increase P nutrition in pulses (Table 3). The most efficient bacteria having P solubilization efficiency include *Bacillus*, *Pseudomonas* and *Rhizobium*. Fungi like *Aspergillus* and *Penicillium* can also convert insoluble phosphorus to soluble forms. Alikhani et al. [94] reported that amongst the rhizobial groups, *Rhizobium leguminosarum* bv. *viciae* exhibited highest inorganic P solubilization. Other inorganic P solubilizers include *Sinorhizobium meliloti*, *Rhizobium leguminosarum* bv. *phaseoli*, *Mesorhizobium ciceri* and *Mesorhizobium mediterraneum*. Mineral phosphate solubilization (MPS) by PSMs is due to the lowering of the pH of the medium either by H^+^ extrusion or due to excretion of low molecular weight organic acids such as gluconic acid which chelates the cations bound with phosphate [95]. In Gram-negative PSMs, extracellular oxidation of glucose to gluconic acid via quinoprotein (pyrroloquinoline quinine, PQQ) glucose dehydrogenase (coded by *gcd* gene) is suggested to be a major mechanism for MPS under P starvation [96]. However, glucose dehydrogenase is an inducible enzyme and the P-solubilizing capacity is adversely affected by the presence of organic acids such as succinate and malate. Inoculation with PSM and PGPR together with mineral phosphorus increases the efficiency of P fertilizer utilization and reduces P application by 50% without any significant reduction of grain yield in plants [97]. On the other hand, a large pool of organic P in most soils is as high as 80% of total P, which is not readily available to plants. Several PSMs capable of producing extracellular enzymes like phosphatase, phytase, etc. can hydrolyze organic P compounds. Thus, PGPR is an integral component of soil-P cycle, playing an important role in solubilization as well as mineralization of P, and transfer P between different soil fractions (between inorganic and organic P pools). In addition to PSM, P uptake is influenced by the association of arbuscular mycorrhizal fungi with roots and these processes occur as a natural response of plants to P deficiency. Arbuscular mycorrhiza can explore available P in the surrounding soil with the aid of hyphae [98] and can solubilize the inorganic phosphates as well as mineralize the organic P [99].

### 2.3. Production of Plant Growth Regulators (Hormones)

Many PGPRs have the ability to produce phytohormones that regulate plant growth. The prominent plant growth regulators and their analogues are auxins, cytokinins, and gibberellins which may modify root system architecture (RSA) [101,102,103]. These phytohormones affect physiological and morphological processes of plants at a very low concentration [104]. They can also change growth pattern and result in bigger and branched roots with a greater surface area. As a result, plants are able to access more nutrients from soil. Besides Nod factor signaling in legume-rhizobial symbiosis, phytohormones are known to play an important role in proper symbiotic development [105].

Auxin is an important group of hormones, which influence plant development through organogenesis, tropic responses, cellular responses such as cell expansion, division, and differentiation, as well as gene regulation [106]. These hormones regulate rhizobial infection, infection thread progression and formation of nodule primordia during early nodulation [107,108,109]. Variety of auxins like IAA (indole-3-acetic acid), IBA (indole-3-butyric acid), IPA (indole-3-pyruvic acid), tryptophol (TOL) and ILA (indole lactic acid) are produced by PGPRs. Out of these, IAA is an essential auxin produced by *Alcaligenes*, *Azospirillum*, *Pseudomonas*, *Pantoea*, *Rhizobium* and *Enterobacter* in the presence of L-tryptophan as a precursor. However, the pure culture of fluorescent *Pseudomonas* sp. produces IAA both in the presence and absence of tryptophan. IAA is also present in the nodules of legumes in much higher quantity than in the roots [105] and auxin accumulation in nodules could be derived from rhizobia. The rhizobial production of IAA in legumes is induced by plant flavonoids [110]. The role of IAA in plant-microbe interactions varies from phytostimulation and pathogenesis, as well as the degradation of aromatic amino acids [111]. The inoculation of IAA-producing *Pseudomonas thivervalensis* induces plant growth at a low cell concentration (10^5^ CFU mL^−1^), however, high cell load (>10^6^ CFU mL^−1^) is proved to be inhibitory [112]. The production of IAA by *Azospirillum*, *Agrobacterium*, *Pseudomonas* and *Erwinia* increases seedling root length, root hairs, root branching and root surface area [113]. IAA producing *Rhizobium* strains showed enhanced lateral root development and increased nodulation [114] with delayed nodule senescence [115]. On the other hand, IAA^-^ deficient mutants of *Bradyrhizobium elkanii* USDA 31 showed a reduced number of nodules in soybean [116]. Failure to develop a mutant for IAA production indicates multiple pathways involved in the production of this hormone, and IAA can be produced via both tryptophan—dependent and tryptophan—independent pathways. In tryptophan dependent pathways, at least five different pathways such as indole-3-acetamide (IAM), indole-3-acetonitrile (IAN), indole-3-pyruvate (IPyA), tryptamine (TAM) and tryptophan side-chain oxidase (TSO) pathways are reported, in which the source of tryptophan can be either degrading roots or bacterial cell exudates [111]. IPyA pathway is linked to rhizosphere fitness, whereas IAM route is associated with pathogenesis [117].

Cytokinins (CK) are purine derivatives characterized by their potential to promote cell division (cytokinesis), cell enlargement and tissue expansion in the plant. Its production has been documented in *Azotobacter*, *Azospirillum*, *Rhizobium*, *Bacillus*, *Burkholderia*, *Klebsiella*, *Paenibacillus* and *Pseudomonas* species [118,119,120,121,122,123]. Cytokinins increase root surface area through the enhanced formation of adventitious and lateral roots, and affect apical dominance, axillary bud growth and leaf senescence. CKs are also involved in signal mediation from roots to shoots under environmental stresses [124]. The cytokinin producing PGPRs also affect the auxin/cytokinin ratio, which in turn regulates plant root architecture [124]. Cytokinins enhance plant growth in soybean, rapeseed and other crops [121,122,123,125]. These are also known to play a vital role in rhizobial infection and nodule differentiation in legumes [126]. Nod^-^ mutant of *Rhizobium* harboring constitutive trans-zeatin secretion (*tzs*) gene mimics the morphogenetic effects of Nod factors and stimulates expression of early nodulin gene (*ENOD2*) in *Medicago sativa* [127]. Strains of *Sinorhizobium* sp. and *Mesorhizobium loti* produce four different types of CKs *viz*., nucleotides (CK-NT), ribosides (CK-RB), free bases (CK-FB) and methyl-thiol CK (CK-MET). The CK-MET is the predominant cytokinin, however, CK-FB is the most biologically active form secreted out [128]. It has been shown that exogenous application of low level of cytokinins was stimulatory, while elevated concentration reduced nodule formation in soybean [129]. The cytokinin receptor mutant phenotype of *Medicago truncatula* and *Lotus japonicus* produced defective nodules [130,131]. Analysis of wild-type and Fix^-^ *sym33* (gene encoding transcription factor IPD3/CYCLOPS regulates infection process and nodule differentiation), as well as *sym40* (gene coding for EFD transcription factor that negatively controls nodulation) mutants of pea revealed a low level of trans-zeatin riboside in mutant nodules, suggesting the role of plant CKs in infection thread formation and bacteroid differentiation [132].

Use of cytokinin producing *Penibacillus polymyxa* affects abscisic acid (ABA) signaling of plants or rhizobia-elicited nodulation [133]. The cytokinin–ABA antagonism is the result of metabolic interactions due to their common biosynthetic origin. The inoculation with cytokinin producing bacteria stimulates shoot growth and magnifies ABA content; thus, eliciting stomatal closure under drought conditions [134]. Inoculation of *Arabidopsis thaliana* with *Azospirillum brasilense* Sp245 increased the plant’s ABA content and helped in stress alleviation [135]. Similarly, *Pseudomonas putida* H-2-3 inoculation reduced stress induced ABA accumulation in soybean plant [136]. A low level of endogenous ABA promotes nodulation efficiency and nitrogen fixation. The enhanced nitrogen fixation is correlated with decreased nitric oxide (NO) production in root nodules without concomitant increase in *nifH* gene expression [137]. It was reported that the exogenous ABA application after rhizobial inoculation suppressed nodulation, while ABA content lower than the normal enhanced nodule formation in *Lotus japonicus* [138]. Studies suggest that ABA induces nodule senescence [139,140].

Gibberellins (GAs) are tetracyclic diterpenoids that regulate germination, stem elongation, flowering and fruiting in plants [141]. Production of GAs by *Achromobacter*, *Acinetobacter*, *Azospirillum*, *Agrobacterium*, *Azotobacter*, *Bacillus*, *Herbaspirillum*, *Gluconobacter*, *Pseudomonas* and *Rhizobia* is well documented [142,143,144,145]. Inoculation of *Azospirillium* sp. reversed rice dwarfism [146] by metabolizing inactive GA precursors into *in planta* active gibberellins [147]. The presence of cytochrome p450 monooxygenase gene cluster involved in GA biosynthetic pathway is reported in *Rhizobium* NGR234 [148] and *Bradyrhizobium japonicum* [149]. The genomic analysis of *Bradyrhizobium japonicum* USDA 110 provided first evidence for the role of diterpenoid operon in GA biosynthesis [150]. Tatsukami and Ueda [151] found that GA synthetic genes are distributed in rhizobial species *viz*., *Mesorhizobium loti*, *Bradyrhizobium japonicum*, *Sinorhizobium* (*Ensifer*) *fredii* and *Rhizobium etli* that inhabit determinate nodules. They observed increased number of nodules in *Lotus japonicus* with GA^-^ deficient *Mesorhizobium loti* mutant and suggested that the putative rhizobial GA possibly regulates optimal N fixation and prevents delayed infection. The low concentration of GA (0.001 mM) promotes nodule formation, while high levels of GA inhibit infection thread formation in pea [152]. GAs differentially influence infection thread formation in root epidermis and nodule organogenesis in cortex cells of legume root nodules. GA^-^ deficient root phenotypes of pea reduced nodule initiation and development by producing more ethylene, which negatively affects nodule formation [152]. The GA^-^ mutant line (*na-1*) showed few underdeveloped nodules, smaller bacteroids with broken peribacteroid membranes that showed reduced nitrogen fixation [152,153]. The application of bioactive GA_3_ significantly increased the number of nodules compared to wild type [153]. It was also observed that a reduced nodule number in *Lotus japonicus* and *Medicago truncatula* with application of GA biosynthesis inhibitors was due to disruption in DELLA proteins (transcriptional activator of GA signaling) [154,155,156]. Changes in the expression of early nodulation gene in DELLA^-^ deficient pea could be due to disruption in lipo-chitooligosaccharide (LCO) or Nod factor signaling [153,157]. Expression analysis of pea plants treated with bioactive GA_3_ showed a negative effect of GA on the nodule senescence [158]. This study revealed that the stimulatory effect of GA application is associated with the down regulation of senescence-associated genes (encoding cysteine proteases 1 and 15a, thiol protease, bZIP transcription factor, 1 aminocyclopropane-1-carboxylate (ACC) synthase, ACC oxidase, and aldehyde oxidase). It was also observed that GA treated plants decrease senescence zone, increase nitrogen fixation zone, nodule size, and stimulate meristem bifurcation.

Ethylene is another key phytohormone which evokes physiological responses in plants at low concentrations. However, elevated levels of ethylene suppress shoot and root growth as well as inhibit nodule development by suppressing the infection thread formation [159,160]. The production of IAA by PGPR activates ACC (1-aminocyclopropane-1-carboxylate) synthase, leading to the production of ACC, which is an ethylene precursor in plants [161]. Certain strains of rhizobia capable of producing ACC deaminase can deaminate ACC to ammonia and α-ketobutyrate, which in turn can reduce the level of ethylene’s inhibition on root elongation [162]. This process can increase nodule number, nitrogen content and plant growth [163]. *Mesorhizobium* strains expressing exogenous ACC deaminase activity improved nodulation ability in chickpea [164]. The genomes of *Rhizobium leguminosarum* bv. *viciae* 128C53K [160], *Bradyrhizobium japonicum* USDA110 [150], *Mesorhizobium* sp. MAFF303099 [165], and *Mesorhizobium ciceri* bv. *biserrulae* WSM1271 [166] are reported to have a structural gene (*acdS*) encoding for ACC deaminase. Moreover, in *Rhizobium*, NifA (positive regulator of *nif* gene) regulated *acdS* expression associated with decreased rate of nodule senescence and increased amount of nitrogen fixation [167]. On the other hand, AcdR (leucine responsive regulatory protein) located in upstream of *acdS* gene regulated *acdS* expression that facilitates nodule formation [162]. Like rhizobia, ACC deaminase producing rhizobacteria can reduce ethylene inhibition and plant growth under biotic and abiotic stress conditions (Table 4). In addition to plant growth promotion and root system architecture, the phytohormones produced by PGPRs are involved in defense signaling network through jasmonate and salicylic acid pathways [168]. Although the synthesis of phytohormones by microbes is well documented, their role in the modulation of plant hormone balance is not fully understood.

## 3. Influence of PGPR Strains on Plant Growth Promotion and Nutrient Uptake

Plant growth-promoting rhizobacteria either alone or in combinations can improve the nutrient use efficiency, thus reducing the application of chemical fertilizers. Combined inoculation of rhizobia and rhizobacteria showed a positive effect on root nodulation and growth in legumes (Table 5). The basic mechanisms involved in this synergistic activity are by altering the host’s secondary metabolism and/or eliminating competition of rhizobia with deleterious microorganisms for colonization of the plants. Alteration in the flavonoid metabolism was another mechanism of synergistic activity of PGPR and rhizobia. Increase in plant yield with PGPR inoculation is attributed to improved root development that facilitates water and nutrient uptake [17,92,182]. Organic acid secretions by PGPRs via proton pump through ATPase [183] can also cause acidification of rhizosphere, which in turn increases the plant uptake of mineral nutrients such as Ca, K, Fe, Cu, Mn and Zn [184]. Inoculation with *Azospirillum* has shown to modify root morphology by increasing finer roots (with greater surface area and lower C costs to plants), root hair density, root branching and conferred greater tolerance to drought stress in common bean [185] and soybean [182]. *Azospirillum* improved root nodulation by creating additional sites for rhizobial root infection [186] as well as induced *nod* genes in *Bradyrhizobium japonicum* USDA 110 at lower–density inoculum through inter-species quorum sensing (QS) communication [187]. PGPRs also alleviate salt and drought stress by altering physiological and molecular processes in plants. Enhanced nutrient uptake and amelioration of adverse effect of salt stress in soybean have been observed with *Bacillus firmus* SW5 inoculation [19]. This strain has significantly boosted proline, glycine betaine content, antioxidant activities and stress-responsive gene expression (*GmVSP*, *GmPHD2*, *GmbZIP62*, *GmWRKY54*, *GmOLPb*, *CHS*) besides promoting root system architecture. Upregulation of *AUX/IAA1* (transcriptional repressor of auxin responsive gene), *TaCTR*_1_ (regulatory component of the ethylene signaling pathway) and *TaDREB*_2_ (dehydration responsive element binding2) genes with inoculation of PGPR under salt and drought stress conditions has also been demonstrated [188]. Tripartite symbiosis of rhizobial and arbuscular mycorrhizal fungi with legumes improved N and P uptake. Transcriptomic analysis in soybean revealed that rhizobial nodulation was enhanced with AM fungi colonization. High transcript levels of genes encoding for endo-ß-1-4-glucanase (responsible for cell wall degradation during root nodule formation), early nodulin and carbonic anhydrase (helps in nodule development) in rhizobial-AM symbiosis suggested the contribution of AM fungal colonization to biological nitrogen fixation [189]. Our recent study showed that chickpea seeds inoculated with culturable endophytic fungi (*Piriformospora indica*) and *Mesorhizobium ciceri* had a synergistic effect with nodulation and nutrient uptake [92]. The use of antibiotic producing actinobacteria as PGPR could offer a competitive advantage over other microbial communities. The inoculation with *Streptomyces* species enhanced mesorhizobial nodulation and plant growth in chickpea under field conditions [190]. Tokala group [191] observed that dual inoculation of plant-growth promoting *Streptomyces lydicus* WYEC 108 with *Rhizobium leguminosarum* enhanced nodulation and nitrogen fixation in pea. This study showed that *Streptomyces lydicus* colonizes the root hairs of pea plants and helps in rhizobial infection, root nodule initiation and bacteroid differentiation.

## 4. Molecular Techniques Used in PGPR Study

For a long time, research has focused on various biochemical and inoculation-based methods to study PGPR, but with recent advances in molecular technologies, the huge amount of genomic, metagenomic, transcriptomic and proteomic data are made available on the worldwide web. Genomic analysis of PGPRs can be divided into two broad categories; namely, (a) whole-genome sequencing analysis of PGPR species where the entire chromosome and plasmid are sequenced and annotated (Table 6), and (b) partial/targeted genome or specific gene sequence analysis where a part of the genome is studied and used for characterization and comparison.

Whole-genome analysis using next-generation sequencing (NGS) gives a detailed account of an organism’s genetics. The most popular gene in this category is 16S ribosomal DNA/RNA, which bears a unique marker of identification of PGPR at the genus level. 16S–23S intra genomic spacer (IGS) has also been targeted for species level identification of PGPRs. The repetitive sequence-based PCR (rep-PCR), which is based on amplifying and sequencing of highly conserved inverted repeats has been performed by different research groups. These inverted repeats can be divided into two categories, namely repetitive extragenic palindromic (REP) elements and enterobacterial repetitive intergenic consensus (ERIC) sequences. Along with these, the 154 bp BOX element is also used to characterize genomes. All three methods, like ERIC-PCR, REP-PCR and BOX-PCR, are efficient to study the genetic diversity of PGPRs. Restriction fragment length polymorphism (RFLP) analysis of 16S rRNA gene was used to group PGPRs (ARDRA-amplified ribosomal DNA restriction analysis of 16S rDNA). A multi-locus sequence analysis (MLSA) of several housekeeping genes such as *atpD*, *recA*, *rpoA*, *rpoB*, *thrC*, *dnaK*, *dn*aJ, *gln*II, *gap*, *gln*A, *gltA*, *gyrB* and *pnp* is used for strain typing [207,208]. Arbitrary primers are used to amplify genome sequences in a random fashion, referred to as random amplified polymorphic DNA (RAPD) analysis. In addition, the amplification of specific genes (for instance 16S or 16S–23S (IGS amplicons)) coupled with secondary analysis methods like restriction profiling and denaturing gradient gel electrophoresis (DGGE) to deduce the groupings formed by mis-matches in restriction sites or difference in GC content of genomes has also been studied (Table 7).

Biological nitrogen fixation is one of the most important growth promoting mechanisms of rhizobacteria with primary involvement of *nif* genes coding for nitrogenase enzyme. While the nitrogenase enzyme is collectively coded by three *nif* genes, namely *nifH*, *nifD* and *nifK*, most of the studies are based on *nifH* PCR and sequencing. Phosphate solubilization by PGPRs involves the secretion of gluconic acid, which requires the enzyme glucose dehydrogenase and its cofactor PQQ [228]. PQQ is encoded by pqq operon which consists of 6 core genes: *pqq A*, *B*, *C*, *D*, *E* and *F*. Besides these, PGPRs produce several phytohormones like IAA, auxins, cytokinins and abscisic acid. Genes involved in IAA production include *ipdC*, *amiE* and *nhase*, but for auxin production, *aec* (auxin efflux carrier) gene is studied (Table 8). For iron sequestering, PGPRs produce siderophores which require the upregulation of *sid* gene. *Pseudomonas* employs a membrane receptor coded by *pup*A gene for the transport of iron-complexed siderophore back into the cell. NtEXP is another plant growth promoting gene which encodes expansin proteins and *acc* (ACC deaminase) gene is also implicated in promoting plant growth and strengthening defense mechanisms by inhibiting excessive ethylene production.

## 5. Conclusions and Prospects

The significance of legumes for improvement and sustenance of soil fertility has been known since crop domestication. Mixed cropping, intercropping and crop rotations of non-legumes with legumes have been employed to capitalize on the biological nitrogen fixation. Besides the natural association between N fixing rhizobia and legume crops, other beneficial rhizobacteria have been used as biofertilizers, phyto-stimulators and biopesticides for enhancing plant growth and soil health, and imparting stress tolerance to plants. However, growth promotion influenced by PGPRs under in vitro conditions needs to be confirmed under in situ conditions. The strain efficacy is usually related to the establishment and population density of the introduced strain in the rhizosphere. Hence, an in-depth study to predict their colonization potential, establishment and plant response under field conditions is essentially required. Diverse microbes form natural colonization with legume roots are known to help in nutrient acquisition and disease protection. Comprehensive information on the impact of potential PGPRs on the resident rhizosphere microbial community *vis-a-vis* the interactive effect of natural microbial community with the introduced PGPRs is required to delineate their rhizosphere competency and functional potential. The type and kind of molecules in root exudates secreted by plants determine the rhizosphere microbial diversity, in which only a fraction of PGPRs are culturable. Therefore, the identification of novel unculturable PGPRs using high throughput sequencing methods and devising strategies to improve their cultivation efficiency needs to be undertaken. Various environmental factors, plant genotypes and soil types can affect PGPR performance under field conditions. An understanding of genetic variation in beneficial host-PGPR interactions can be integrated in breeding varieties with heritable plant-associated microbial community for improving legume productivity.

## Figures and Tables

**Table 1:** Influence of plant growth promoting rhizobacteria on growth of legume crops.

Crop	Microbes	Beneficial Effects	References
*Cicer arietinum* (Chickpea)	*Pseudomonas aeruginosa*, *Pseudomonas alcaligenes*, *Pseudomonas fluorescens* BHUPSB06, *Pseudomonas jessenii* PS06	Enhanced acquisition of P and Fe, effective symbiosis with *Mesohizobium*	[23,24,25]
	*Pseudomonas alcaligenes*, *Bacillus pumilus*	Increase in shoot dry mass, pod number, nodulation, chlorophyll content, N, P and K content	[24,25]
	*Azospirillum lipoferum* FK1, *Azospirillum brasilense*	Improved nodulation and growth	[19,26]
	*Azotobacter*	Increase in plant–rhizobial symbiosis, biomass, grain yield, N content	[27]
*Lens culinaris* (Lentil)	*Bacillus megaterium* *Kurthia* sp. LK786, *Pseudomonas diminuta* LK884	Enhanced symbiotic effect of *Rhizobium leguminosarum* and improved plant growth	[28,29]
	*Pseudomonas* sp.	Enhanced symbiotic effect of *Rhizobium leguminosarum* and improved plant growth	[30]
	*Proteus vulgaris*	Increased nodulation potential when given in combination with *Rhizobium leguminosarum* L-12-8	[31]
*Vigna radiata* (Green gram)	*Bacillus subtilis*, *Bacillus megaterium*	Increase in dry matter and N and P uptake	[32,33]
	*Pseudomonas putida* GRP3A	Stimulated iron uptake	[34]
	*Pseudomonas* sp.	Increase in plant height and improved root development	[34,35,36]
*Cajanus cajan* (Pigeonpea)	*Bacillus subtilis* AF1, *Bacillus cereus* BS03	Increase in shoot, root length, nodulation and biomass	[37,38]
	*Pseudomonas* spp., *Pseudomonas aeruginosa* RRLJ	Significant increase in plant growth and nodulation occupancy of *Rhizobium*	[38,39]
	*Azotobacter chroococcum* A41, *Bacillus megaterium* MTCC 453, *Pseudomonas fluorescens* MTCC9768.	Improved plant growth and yield	[40]
*Arachis hypogaea* (Groundnut)	*Bacillus*, *Pseudomonas fluorescens*	Enhanced synergistic activity of rhizobia for nutrient uptake and plant growth	[41,42]
*Glycine max* (Soybean)	*Bacillus amyloliquefaciens* LL2012, *Bacillus subtilis*	Enhanced symbiotic capacity of *Bradyrhizobium japonicum*	[31,43]
	*Azospirillum brasilense* Sp7, *Azospirillum lipoferum* CCM3863	Efficient symbiosis with *Bradyrhizobium japonicum* and enhancement in root growth and shoot dry matter	[44]
	*Pseudomonas cepacia*	Enhanced synergistic activity with *Bradyrhizobium japonicum* TAL-378 resulted in overall improved plant growth	[45,46]
*Phaseolus vulgaris* (Common bean)	*Bacillus megaterium*	Increased nodulation, shoot dry weight, nodule dry weight and chlorophyll content	[47]
	*Paenibacillus polymyxa* DSM 36 and Loutit (L)	Increased symbiotic efficiency of *Rhizobium tropici*	[48]
	*Azospirillum brasilense*, *Azospirillum lipoferum* S21	Enhancement of nodulation and N_2_ fixation activity of *Rhizobium*	[49,50]
	*Pseudomonas monteilii*, *Pseudomonas fluorescens* P93	Synergistic effect of *Rhizobium pisi* leading to increased nodulation	[48,51]
*Vicia faba* (Faba bean)	*Azospirillum brasilense*, *Azospirillum lipoferum* SM1, *Azospirillum brasilense*	Increase in growth of root, shoot and improved nodulation	[26,52]
	*Azotobacter chroococcum* H23, *Azotobacter vinelandii* ATCC12837 and Dv42	Increased nodulation, dry mater and total N content	[53]
	*Pseudomonas aeruginosa*, *Pseudomonas putida* TK3, *Serratia marcescens* BM1 *Serratia liquefaciens* BM4, *Xanthobacter autotrophicus* BM3	Increase in the phytoremediation potential Increase in shoot dry weight, number of pods per plant and nodule dry weight	[52,54]
	*Pseudomonas fluorescens*, *Pseudomonas alcaligenes* PsA15, *Pseudomonas denitrificans* PsD6	Increase in fresh and dry weight, root and shoot length, number of leaves per plant	[55,56]
	*Bacillus polymyxa* BcP26, *Mycobacterium phlei* MbP18, *Cellulomonas* sp. 32	Increase in root and shoot growth, nodulation, increase in N and P content	[55]
*Phaseolus vulgaris* (French bean)	*Pseudomonas lurida* NPRp15, *Pseudomonas putida* PGRs4	Increased root and shoot dry weight, nodulation, nutrient uptake, pod yield	[57]
*Vigna unguiculata* (Cowpea)	*Pontibacter niistensis* NII-0905	Increase in root number, root length, shoot length and dry biomass	[58]

**Table 2:** Range of nitrogen fixed by associations of important legume crops with rhizobia.

Legume Crop	Associated Rhizobial Strains	Amount of N Fixed (kg ha^−1^)	Method of Estimation	Reference
*Cicer arietinum*	*Bradyrhizobium ciceri* bvs. CP31, CP36	19–24	^15^N isotope dilution	[75]
	*Rhizobium* sp.	15–32	^15^N natural abundance	[76]
*Cajanus cajan*	*Rhizobium* sp. IHP114	13–69	N Difference	[77]
*Vigna radiata*	*Bradyrhizobium japonicum* 542	116	N Difference	[78]
*Lens culinaris*	*Rhizobium leguminosarum* bv. *viciae* su391	25	^15^N isotope dilution	[79]
	*Rhizobium leguminosarum*	0–105	^15^N isotope dilution	[80]
	*Rhizobium* sp.	37–55	^15^N natural abundance	[81]
	*Bradyrhizobium* sp.	82	N Difference	[82]
*Phaseolus vulgaris*	*Rhizobium leguminosarum* bv. *phaseoli*	24–39	^15^N isotope dilution	[83]
	*Rhizobium leguminosarum* bv. *phaseoli*	11–165	^15^N isotope dilution	[84]
*Phaseolus vulgaris*	*Rhizobium phaseoli*	78.7	^15^N isotope dilution	[85]
*Vicia faba*	*Rhizobium leguminosarum* bv. *viciae*	76–125	^15^N isotope dilution	[75]
	*Bradyrhizobium* sp.	210	N Difference	[82]
	*Rhizobium phaseoli*	3.8	acetylene reduction	[85]
*Pisum sativum*	*Rhizobium leguminosarum* bv. *viciae* su-391	34–112	^15^N isotope dilution	[79]
	*Bradyrhizobium* sp.	128	N Difference	[82]
	*Rhizobium leguminosarum* bv. *viciae*	31–107	^15^N isotope dilution	[75]
*Arachis hypogaea*	*Rhizobium* sp.	186	N Difference	[86]
	*Bradyrhizobium* sp.	150–200	^15^N isotope dilution	[87,88]
*Glycine max*	*Bradyrhizobium japonicum*	102.9	^15^N isotope dilution	[85]
	*Bradyrhizobium japonicum*	25.6	acetylene reduction	[85]
	*Bradyrhizobium* sp.	108–152	^15^N isotope dilution	[88]

**Table 3:** P nutrition of legume crops mediated by plant growth-promoting rhizobacteria.

PGPR	Crop	Reference
*Pseudomonas aeruginosa*	*Cicer arietinum*	[100]
*Pseudomonas alcaligenes*, *Bacillus pumilus*	*Cicer arietinum*	[24]
*Bacillus megaterium*	*Lens culinaris*	[28]
*Bacillus megaterium*	*Phaseolus vulgaris*	[47]
*Pseudomonas fluorescens*	*Arachis hypogaea*	[41]
*Pseudomonas lurida-NPRp15* and *Pseudomonas putida*-*PGRs4*	*Phaseolus vulgaris*	[57]
*Bacillus subtilis*	*Vigna radiata*	[32]

**Table 4:** ACC-deaminase producing PGPR strains promoting growth and stress alleviation in legume crops.

Legume Crop	Associated PGPR	Effect	Reference
*Cicer arietinum*	*Serratia proteamaculans* J119	Improved root and shoot growth, nodulation, grain yield	[169]
	*Mesorhizobium ciceri* LMS1	Increase in nodulation and plant growth	[164]
	*Mesorhizobium*	Improved plant growth under salinity stress	[170]
*Lens culinaris*	*Bacillus cereus*, *Pseudomonas* sp.	Promoted plant growth under axenic conditions	[171]
*Vigna radiata*	*Pseudomonas putida*, *Pseudomonas fluorescens*, *Bradyrhizobium japonicum*	Root elongation, increase in nodule number, nodule fresh and dry weight	[172]
	*Pseudomonas fluorescens*, *Pseudomonas syringae*, *Rhizobium phaseoli*	Significantly reduced salinity stress and increase plant growth	[173]
*Pisum sativum*	*Rhizobium leguminosarum* bv. viciae 128C53K	Increased nodulation	[162]
	*Arthrobacter protophormiae*	Increased plant tolerance to salt stress and improved plant growth	[174]
	*Pseudomonas brassicacearum* Am3, *Pseudomonas marginalis* Dp1,	Enhanced nutrient uptake	[175]
	*Pseudomonas fluorescens*, *Pseudomonas putida*,	Reduced drought stress on plant	[176]
	*Variovorax paradoxus* 5C2	Improved growth, yield and water use efficiency of drought stressed plants	[177]
*Glycine max*	*Pseudomonas* sp.	Increased plant growth and reduced plant fungal disease	[178]
*Arachis hypogaea*	*Pseudomonas* sp.	Enhanced growth, yield and nutrient uptake	[41]
	*Pseudomonas fluorescens* TDK1	Enhanced resistance to saline stress	[179]
*Cyamopsis tetragonoloba* (Cluster bean)	*Pseudomonas* sp.	Improved nodulation and plant growth	[180]
*Vigna unguiculata*	*Pseudomonas* sp.	Improved plant growth under salt stress	[181]

**Table 5:** Effect of co-inoculation on plant growth and development.

Co-Inoculated Strains	Legume Plant	Positive Effects on Plant Growth Parameters	Reference
*Rhizobium leguminosarum* bv. *viciae*, *Pseudomonas* sp. (PSB), *Pseudomonas* sp. (PGPR)	*Lens culinaris*	81% increase in nodule number, 57% increase in nodule dry weight and 15% improvement in grain yield *	[192]
*Rhizobium CRM 6*, *Bacillus polymyxa* (PSB), PGPR (KB 133)	*Vigna radiata*	110% increase in nodule number, 121% increase in nodule weight and 44% increase in grain yield *	[193]
*Bacillus amyloliquefaciens* LL2012, *Bradyrhizobium japonicum*	*Glycine max*	50% increase in shoot dry weight and 40% increase in root dry weight #	[43]
*Mesorhizobium ciceri* CH-1233, *Pseudomonas* sp. LK884	*Cicer arietinum*	56% increase in nodule number, 100% increase in nodule dry weight, and 15% increase in grain yield *	[194]
*Bradyrhizobium* sp., *Serratia marcescens*, *Trichoderma harzianum*	*Arachis hypogaea*	115% increase in nodule number, 94% increase in nodule dry weight and 41% increase in grain yield *	[195]
*Rhizobium*, *Azotobacter chroococcum*	*Cajanus cajan*	248% increase in nodule number, 100% increase in nodule dry weight and 92% increase in N fixation, and 19% increase in grain yield #	[196]
*Rhizobium*, *Pseudomonas fluorescens*	*Cajanus cajan*	388% increase in nodule number, 267% increase in nodule dry weight and 134% increase in N fixation and 66% increase in grain yield #	[196]
*Rhizobium*, *Bacillus cereus*	*Cajanus cajan*	382% increase in nodule number, 196% increase in nodule dry weight and 116% increase in N fixation and 54% increase in grain yield #	[196]
*Glomus fasciculatum* (VAM), *Rhizobium*	*Cajanus cajan*	19% increase in chlorophyll content, 10% increase in N content and 114% increase in P content #	[197]

* Study performed in field; # Study carried out in pot conditions.

**Table 6:** Whole genome data of PGPR available in worldwide web.

PGPR	Host Plant	Genome Size (Mb)	Reference
*Mesorhizobium ciceri* CC1192	*Cicer arietinum*	6.94	[198]
*Herbaspirillum lusitanum* P6-12	*Phaseolus vulgaris*	4.46	[199]
*Bradyrhizobium yuanmingense* BR 3267	*Vigna unguiculata*	7.90	[200]
*Sinorhizobium fredii* USDA257	*Glycine max*	6.47	[201]
*Bradyrhizobium japonicum* CPAC 15, *Bradyrhizobium diazoefficiens* CPAC 7	*Glycine max*	9.58	[202]
*Stenotrophomonas maltophilia* RR-10	*Oryza sativa* (Rice)	4.66	[203]
*Pseudomonas strain* R62 and R81	*Triticum* sp. (Wheat)	6.00	[204]
*Bacillus amyloliquefaciens* BS006	*Musa* sp. (Banana)	4.17	[205]
*Azospirillum brasilense* CBG497	*Zea mays* (Maize)	6.50	[206]

**Table 7:** Commonly employed molecular techniques to profile the PGPR diversity.

Method	PGPR Community/Source Plant	Reference
16S rDNA sequencing	Rhizobia, *Pantoea agglomerans*, *Exiguobacterium*, *Ensifer*, *Bacillus* sp., *Pseudomonas* and *Leclercia*	[209,210,211,212]
16S-23S IGS sequencing	*Rhizobium leguminosarum bv*. *viciae*, *trifolii*, and *phaseoli*, *Mesorhizobium* populations	[213,214]
REP-PCR, ERIC-PCR DNA fingerprinting	*Mesorhizobia* sp.	[215]
	*Rhizobium meliloti* solates	[216]
	Rhizobia associated with Belgium legumes	[217]
Box PCR	*Mesorhizobium* populations associated from Chickpea	[214]
	Rhizobia associated with common bean	[218]
ARDRA	*Mesorhizobium*, common bean rhizobia	[214,218]
MLSA	*gyrB* (DNA gyrase), *rpoD* (RNA polymerase) of *Pseudomonas*	[217]
	*atpD* (ATP synthase)	[219]
	*gyrB*, *nifK* and *nod* genes of *Mesorhizobium*,	[220]
	*recA* of *Burkholderia* sp.	[221]
RAPD-PCR	*Rhizobium leguminosarum bv*. *viciae* strains, *Azotobacter* and *Trichoderma* strains, *Bradyrhizobium japonicum* strains	[222,223,224]
DGGE	*Azospirillum brasilense* in maize	[225]
	*Acinetobacter* community from wheat Rhizospheric microbial community in pigeonpea	[226,227]

**Table 8:** Genes activated during direct mode of action of PGPR.

PGP Trait	Related Genes	PGPR Strains	References
Nitrogen fixation	*nifH*, *nifD*, *nifK* (nitrogenase iron protein)	*Paenibacillus* sp., *Klebsiella* sp., *Azospirillum* sp., *Burkholderia* sp., *Bacillus* sp., *Mesorhizobium* sp.	[229,230,231]
Phosphate solubilization	*pqqC*, *pqqBCD*, *pqqAB*, *pqqE*, *pqqF* (Pyrrolo Quinoline Quinone Synthase) *gdh* (Glucose Dehydrogenase, cofactor for pqq genes)	*Pseudomonas* sp., *Pseudomonas fluorescens* QAU67, *Pseudomonas putida* QAU90, *Bacillus* sp.	[232]
Siderophores production	*pupa* (siderophore transporter), *sid* (siderophore synthesis), *dhbF* (2,3-Dihydroxy Benzoate synthesis gene)	*Pseudomonas putida* *Bacillus subtilis* AH18 *Bacillus* *licheniformis* K11	[233,234,235]
IAA synthesis	*nhase* (nitrile hydratase), *amd* (amidase), *ipdC* (indole-3-pyruvate decarboxylase), *aec* (auxin efflux carrier protein)	*Rhodococcus erythropolis*, *Pseudomonas putida* *Bacillus subtilis* AH18	[234,236,237]
